# Larotrectinib Treatment of Xanthogranuloma With NTRK1 Gene Fusion: A Case Report

**DOI:** 10.7759/cureus.84609

**Published:** 2025-05-22

**Authors:** Grace Ralston, Michael Humeniuk

**Affiliations:** 1 Internal Medicine, Edward Via College of Osteopathic Medicine, Spartanburg, USA; 2 Medical Oncology, Gibbs Cancer Center, Spartanburg Regional Healthcare System, Spartanburg, USA

**Keywords:** adult xanthogranuloma, larotrectinib, non-langerhans cell histiocytosis, ntrk1 gene fusion, xanthogranuloma, xanthoma disseminatum

## Abstract

Non-Langerhans cell histiocytosis (NLCH) is a broad disorder encompassing different diseases arising from dermal dendritic histiocytes. Xanthogranuloma (XG) is a cutaneous subtype of NLCH that can be present in isolated or multiple locations. If localized, XGs can be treated with surgical resection or radiation; however, if they present more disseminated, they are poorly responsive to traditional chemotherapeutic agents. This case discusses a 26-year-old male who presented with diffuse light brown/tan papules progressively spreading from the bilateral groin axillae to the trunk, pelvis, and eyelids over the course of six months. Histologic evaluation demonstrated findings consistent with XG with immunostaining strongly positive for neurotrophic tyrosine kinase receptor 1 (NTRK1) gene fusion. He was diagnosed as likely having an early form of XG disseminatum before visceral involvement had developed, but given how rare and complex XG diseases are to classify, he might also be considered to have disseminated adult XG. He was started on larotrectinib, an oral tropomyosin inhibitor used to treat solid tumors associated with NTRK gene fusions. Improvement in the lesions was noticeable as early as six weeks, with a successful partial clinical response by four months of treatment, where he ceased to develop new lesions, and the pre-existing lesions became more macular. He eventually discontinued therapy due to a known side effect of weight gain and has continued to maintain his response since. This report demonstrates a positive clinical response of an NTRK1 inhibitor in an adult with NTRK1-positive XGs, providing further evidence that targeted therapy may be beneficial for these otherwise challenging patients.

## Introduction

Histiocytic disorders are rare and complex and are typically divided into Langerhans cell histiocytosis (LCH) and non-Langerhans cell histiocytosis (NLCH) [1].According to the Histiocyte Society’s 2016 revised classification, xanthogranulomas (XGs) are a family of NLCH in the C-group (cutaneous and mucocutaneous histiocytoses) and are then further sub-classified according to their various clinical presentations. NLCH also includes the groups: the R group (Rosai-Dorfman disease and others), the M group (malignant histiocytoses), and the H group (monogenic inherited conditions). Histologically, XGs appear as a cluster of histiocytes that stain positive for CD68 and negative for CD1a and S100, which are more commonly seen in Langerhans cells [2,3]. Clinically, XGs most commonly present either in the first few years of life as cutaneous red or yellow papules that tend to resolve spontaneously and are classified as juvenile XG (JXG) or in adults as solitary persistent nodules classified as adult XG (AXG) [1,2]. Adults can also present with generalized eruptive histiocytosis with crops of flesh-colored or red nodules, or xanthoma disseminatum with brown papules mainly around the eyes or flexor surfaces and heavily associated with visceral involvement [1].Whereas LCH is sometimes found to have BRAF V600E or MAP2K1 mutations, some XG spectrum diseases, including JXG and AXG, can harbor neurotrophic tyrosine kinase receptor 1 (NTRK1) fusions [3].While NTRK1 fusions are only seen in up to 1% of common solid tumors such as colorectal adenocarcinoma or non-small cell lung cancer, some rare cancers such as secretory breast carcinoma can be positive in the majority of cases [4].We present a case using the small-molecule tropomyosin receptor kinase inhibitor, larotrectinib, to treat an adult patient with XGs, positive for a NTRK1 fusion.

## Case presentation

**Figure 1:**
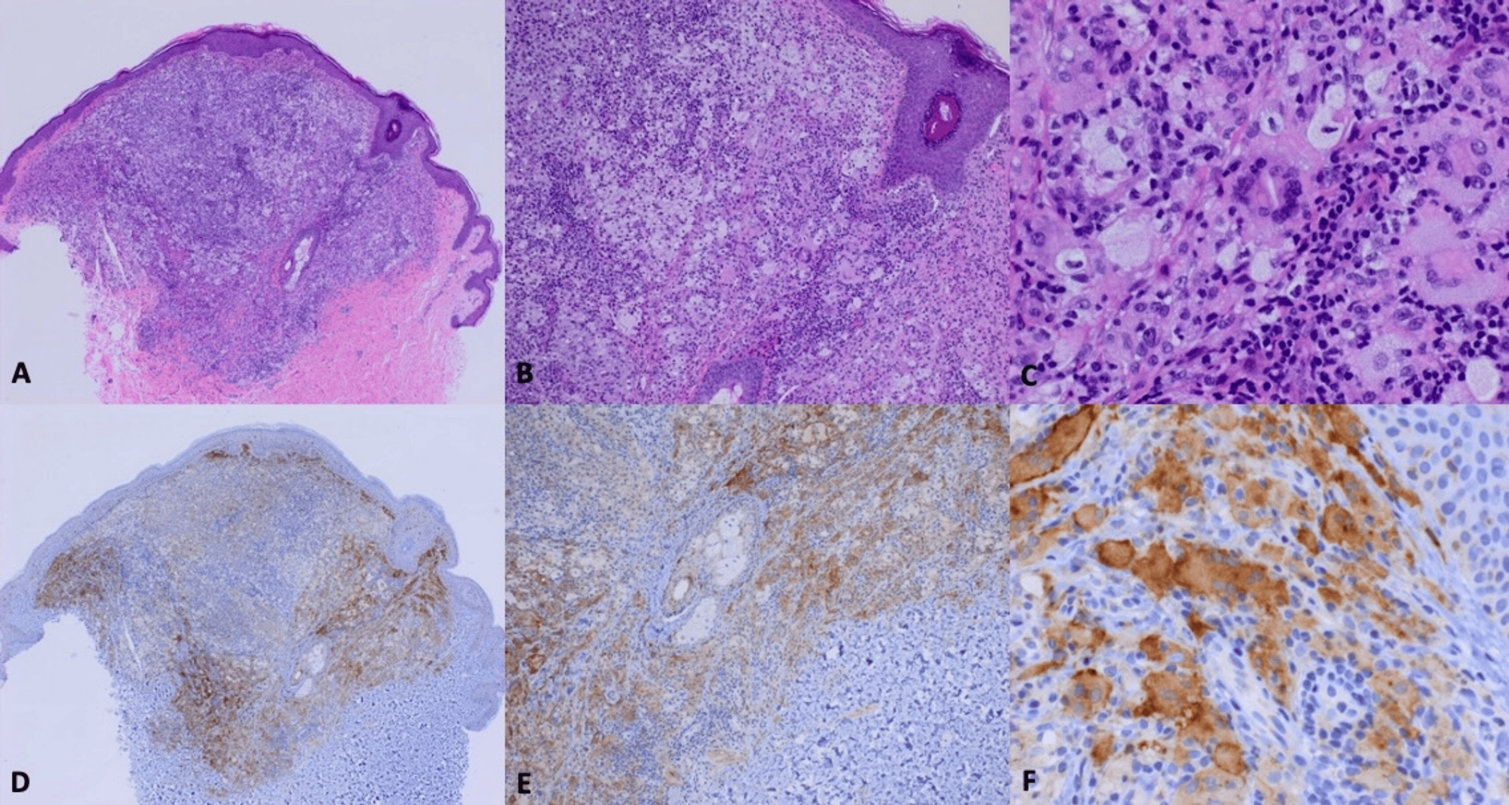
(A, B) H&E stains. (C) High power view of Touton giant cells and multinucleated histiocytes. (D-F) Positive NTRK1 immunostain in some of the histiocytes. H&E, hematoxylin and eosin

A 26-year-old healthy male presented with the progressive spread of multiple 1-4 mm light brown/tan papules originating in the bilateral groin axillae. The nodules were soft, painless, and non-pruritic. Over the course of six months, the lesions spread to the confluent trunk, pelvis, and separately to his eyelids. His physical examination was otherwise unremarkable. A punch biopsy from the axilla demonstrated non-lipidized, foamy, multinucleated histiocytes and a few Touton giant cells, accompanied by superficial dermal lymphocytic infiltration (Figures 1A-1C). Immunostaining was positive for CD68 and negative for S-100 and CD1a, consistent with being an XG. Staining for the BRAF V600E protein product was negative, but the immunostaining for NTRK1 was strongly positive and consistent with a NTRK1 gene fusion (Figures 1D-1F). Unfortunately, further next-generation gene sequencing was not able to be obtained. Whole body PET was negative for any other non-cutaneous sites of disease. Given the patient’s clinical presentation, he was diagnosed as likely having an early form of XG disseminatum before visceral involvement had developed, but given how rare and complex XG diseases are to classify, he might also be considered as disseminated AXG.

**Figure 2:**
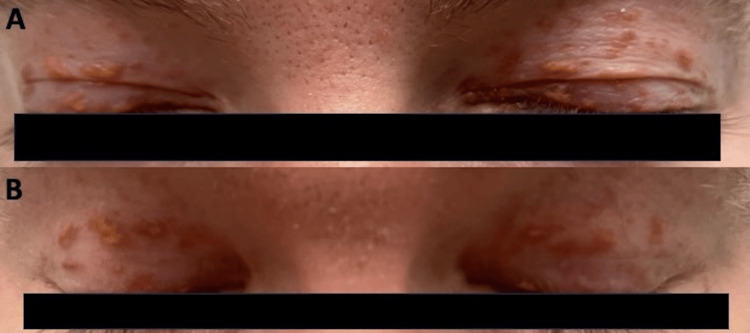
(A) Xanthogranulomas of the eyelids before initiating therapy. (B) 11 months after discontinuing treatment, with noticeable flattening of papules and no new identifiable lesions.

**Figure 3:**
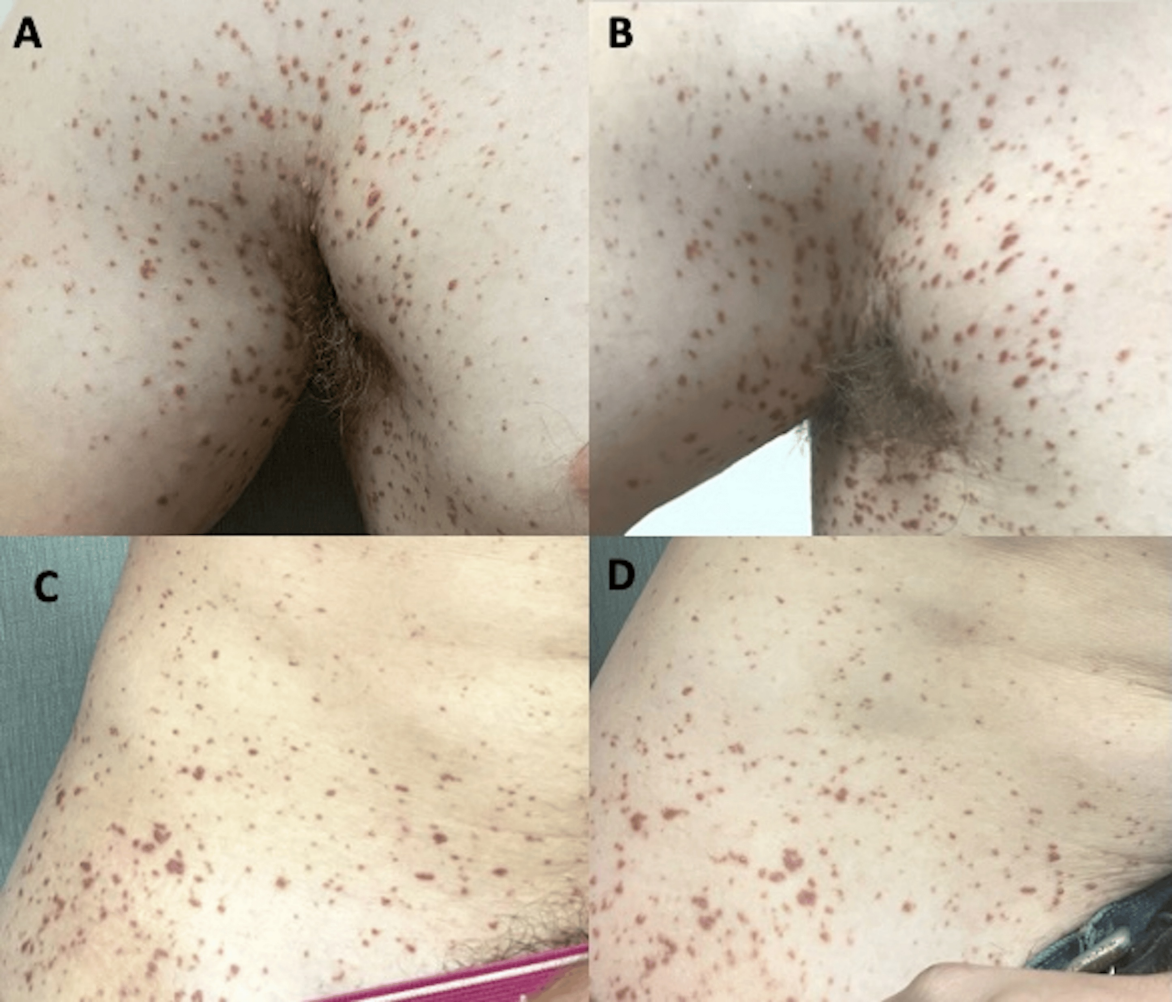
(A) Xanthogranulomas (XG) of the axilla before initiating therapy. (B) 11 months after discontinuing treatment. (C) XGs of the lower abdomen/groin region before initiating therapy. (D) 11 months after discontinuing treatment.

Since his lesions were diffuse and continuously progressing, further therapy was warranted. He was started on the oral tropomyosin kinase inhibitor, larotrectinib, 100 mg twice a day. After two weeks of therapy, he stated that the lesions had stopped growing, and at 6 weeks, some of the papular lesions had begun to flatten, turn more macular, and regress. After four months of therapy, with no new lesions, and the pre-existing lesions continuing to stay pigmented, the patient decided to stop larotrectinib due to having a 5-kg weight gain. At his last documented office visit, over 11 months after drug stoppage, no further progression of his lesions occurred, with some even continuing to flatten and regress (Figures 2, 3). Overall, he had a partial clinical response that was durable while off therapy and now plans, with close observation, for reinitiation of therapy at the time of progression. 

## Discussion

NTRK is a family of tropomyosin receptor kinases, including NTRK1, NTRK2, and NTRK3 [4].NTRK fusions can result in the continuous activation of mitogen-activated protein kinase and phosphoinositide 3-kinase/protein kinase B pathways, both of which play roles in the proliferation and differentiation of multiple cell lineages, including oncologic cell lines [5].While NTRK1 gene fusions are rare, they are still considered a key oncogenic driver, and multiple therapeutics now exist to block this target and arrest cancer growth regardless of the tissue of origin [6,7].

Larotrectinib is an FDA-approved kinase inhibitor indicated for locally advanced or metastatic solid tumors in adults and pediatric populations with NTRK gene fusions and for whom no satisfactory alternative therapies are available [4,7,8].Results from three combined phase I-II trials using larotrectinib across multiple solid tumors, including lung and colon cancers, showed an overall response rate of 80% (95% CI 67-90), including a complete response rate of 16% [4].Responses to larotrectinib were durable, and the progression-free survival at 1 year was 55% [4,5].Larotrectinib is generally well tolerated, with the majority of clinically significant adverse events reported as grade 1 or 2, with less than 5% of patients having a grade 3 adverse event and none deemed treatment-related [4].The most common grade 1 and 2 events were elevated alanine aminotransferase (ALT) and aspartate aminotransferase (AST), fatigue, vomiting, dizziness, anemia, weight gain, and gastrointestinal upset [7]. The most common treatment-related grade 3 events were elevated AST/ALT, dizziness, nausea, anemia, and neutropenia [7]. No grade 4 or 5 events were considered larotrectinib-related, and there were no documented cases of patients with a positive response discontinuing therapy due to an adverse event. Only 15% of patients required a dose reduction [4,7].

The current systemic therapy of XGs is limited and involves the use of immunosuppressive medications such as corticosteroids, cyclosporin, intravenous immunoglobulin, and sometimes cytotoxic chemotherapeutic agents such as vincristine, methotrexate, and doxorubicin [9].This patient with XG had a partial clinical response to larotrectinib within a four-month timeframe with minimal adverse effects other than mild weight gain, a documented side effect of the therapy, and for which he discontinued therapy but was willing to restart when necessary. Evidence for using NTRK1 inhibitors for XGs is extremely limited, with only one other case of disseminated JXG [10] being successfully treated with larotrectinib.In that case [10], the authors state that the patient demonstrated significant improvement in the size and appearance of the JXGs within 2 weeks and did not develop further lesions after initiating therapy.

## Conclusions

In conjunction with the prior case of NTRK1 inhibitor response in JXG, the current report of a response in an adult with XGs gives further evidence that targeted therapy may be beneficial for patients with XG across multiple ages and clinical presentations, harboring NTRK1 fusions. Further study is warranted for these histiocytoses that otherwise have limited options.
